# Sensorineural hearing loss in chronic suppurative otitis media with and without cholesteatoma

**DOI:** 10.1016/S1808-8694(15)30128-2

**Published:** 2015-10-19

**Authors:** Alexandre Fernandes de Azevedo, Daniele Cristine Gomes Pinto, Nicodemos José Alves de Souza, Dirceu Bartolomeu Greco, Denise Utsch Gonçalves

**Affiliations:** a M.S. Otorhinolaryngology. Preceptor of Otorhinolaryngology at the Santa Casa de Misericórdia in Belo Horizonte; b Otorhinolaryngologist, Resident from the Otorhinolaryngology Department -Santa Casa de Misericórdia in Belo Horizonte; c M.S. Otorhinolaryngologist. Head of the Otorhinolaryngology Department - Santa Casa de Misericórdia in Belo Horizonte; d PhD. Full Professor of Internal Medicine - School of Medicine - Federal University of Minas Gerais (UFMG). MD. Advisor of the Postgraduate program in infectology and tropical medicine of the UFMG; e PhD. Adjunct Professor at the Department of Ophthalmology and Otorhinolaryngology and Hearing and Speech Therapy of the UFMG Medical School. Otorhinolaryngologist. Full advisor of the Postgraduate program in infectology and tropical medicine - UFMG. Federal University of Minas Gerais Medical School. Postgraduate program in Health Sciences: infectology and tropical medicine

**Keywords:** colesteatoma middle ear, otitis media suppurative, hearing loss

## Abstract

Sensorineural hearing loss (SNHL) related to chronic suppurative otitis media (CSOM) was studied to clarify the involvement of cholesteatomas in this context. **Aim:** to evaluate SNHL related to CSOM and its association with cholesteatomas, disease duration and patients’ ages. **Methods:** Retrospective analysis of 115 patients with CSOM with and without cholesteatoma submitted to surgical treatment. Inclusion criteria were active unilateral disease, normal contralateral ear and age below 60 years. **Results:** The average age was 26.3 years, 58 males and 57 females. The duration of ear disease was, in average, 12.4 years. The average threshold of hearing was 40 dB in CSOM ear and 22 dB in the normal contralateral ear (P=0.002). CSOM with cholesteatoma occurred in 78 of 115 cases. In the abnormal ear, SNHL was seen in 15 cases, being 6 cases of profound loss, that correlated with adjusted-age (P=0.003) and absence of cholesteatoma (P=0.01), but not with disease duration (P=0.458). **Conclusion:** SNHL occurred in 13% of the patients with CSOM, and was correlated with older age, but not with the presence of cholesteatoma or longer duration of ear disease.

## INTRODUCTION

Chronic otitis media may be considered a public health problem. In the United States, it is estimated that over two billion dollars are spent yearly to treat both acute and chronic ear infections^1^. In developing countries, the prevalence of middle ear infections reaches 72 cases per 1,000 inhabitants and chronic otitis media is the main cause of hearing loss in children^2^. In Brazil, epidemiological studies show an association between chronic otitis media and hearing loss in school age children^3^.

Now, considering suppurative chronic otitis media (SCOM), this may or may not be associated with the development of a cholesteatoma, and conductive hearing loss is the characteristic audiologic alteration^4^.

The association between chronic otitis media and sensorineural hearing loss (SNHL) has been broadly studied and remains a controversial topic. Studies present a frequency that varies from no clinical significance^5^, ^6^, ^7^, ^8^, ^9^ to 10%10. A variation in sample selection could justify this difference. Some studies do not differentiate between SCOM with and without cholesteatoma^5^, ^11^. Others assessed SCOM without considering the presence or absence of cholesteatomas separately^6^, ^7^, ^12^. There is much controversy about the correlation between SNHL with the patient’s age and chronic otitis media duration time^5^, ^6^, ^7^, ^8^, ^9^, ^10^, ^11^, ^12^. The socio-economic environment could also be a possible factor influencing SNHL evolution^2^, ^3^.

The goal of the present investigation was to correlate SCOM associated with SNHL with cholesteatoma with patient’s age and disease duration.

## METHODS

The present study was carried out through the analysis of charts from those patients submitted to otologic surgery because of SCOM with or without cholesteatomas. As for inclusion criterion we considered chronic unilateral otorrhea with a normal contralateral ear in patients with less than 60 years of age. As exclusion criteria, we considered the family history of congenital or acquired SNHL, history of noise exposure, head injury, prior ear surgery, or the possibility of a perilymphatic fistula. The socio-economic statuses of the patients were controlled: having a private health insurance and having been submitted to ear surgery in a hospital that does not belong to the public health care system.

All patients were assessed, treated and followed up by the same professional, with vast experience in otology. In the month prior to the otologic surgery, audiometry exams were carried out in double by two independent professionals and who did not know about the patient’s clinical history or about the presence or absence of cholesteatoma. The exams were carried out in the same equipment and in a sound treated booth. In the audiometry analysis, the air-bone gap considered was of 10 dB to define a conductive hearing loss^13^. In order to define SNHL, we considered a 30dB hearing loss or above for the bone hearing threshold. This cutting point was used in order to rule out the inclusion of those cases that did not represent significant damage to the inner ear^10^. In order to increase the analysis specificity, we assessed the bone hearing loss alone in 4 KHz and the frequency averages of 0.5, 1 and 2 KHz^10^.

We assessed the correlation between SNHL with the following variables: age, ear disease duration and the presence or absence of cholesteatoma. The criterion to define the presence of cholesteatoma was the pathology description of the surgical specimen. In the case of the non-cholesteatomatous disease we considered the criteria of continuous otorrhea, resistant to antibiotic treatment for at least two months, associated to central eardrum perforation, and confirmation of cholesteatoma-free situation during surgery.

The size of the calculated sample was of at least 95 patients, considering the SNHL frequency in patients with SCOM as being of 10%^10^, ^14^. The control used was the contralateral ear (paired) with normal otoscopy. The significance level used was of 5% and the power was of 80%. The null hypothesis formulated was that the presence or absence of cholesteatomas in patients with SCOM did not interfere in the SNHL evolution. This research was classified as being of no risk for the patients (Cap. II, Art. 13) of resolution 196, of October 10, 1996, from the Ministry of Health, which rules clinical research in human beings in Brazil and was approved by the Ethics Committee in Research of the Institution, ETIC registration # 128/04.

## RESULTS

The charts of 115 patients were assessed. Mean age was of 26.3 (SD=15.66), varying between 4 and 59 years, being 58 men and 57 women.

Average hearing threshold was of 40 dB in the ear with SCOM and 22 dB in the normal contralateral ear (P=0.002, CI=14.00-28.48).

Mean duration of the ear disease was of 12.4 (SD=10.9), varying from 1 to 42 years. Table 1 shows the linear regression model to assess SNHL correlation with age and disease duration. We did not observe any correlation with duration (P=0.458), upholding the lack of statistical association when the analysis was stratified for the presence or absence of cholesteatoma. We observed a correlation with the adjusted age (P=0.003), in such a way as that for every 4 years of age increase, there was a reduction of 1 dB in bone conduction, considering a 7% data dispersion and controlling the variable cholesteatoma.

**Table 1:** Linear regression model for patient’s age and ear disease duration influencing the evolution towards sensorineural hearing loss in 115 cases of suppurative chronic otitis media.

Model	Coefficient	Standard Deviation	P value
Constant	9.73	2.54	<0.001
Disease duration	0.10	0.13	0.458
Age	0.25	0.08	0.003

In the ear with SCOM, we observed normal air conduction audiometry in 25 (21.7%) cases, mild hearing loss in 43 (37.4%), moderate in 41 (31.7%) and severe or profound in 6 (5.2%) cases. Considering the bone via, SNHL was seen in 15 (13%) cases, 8 of isolate loss in 4 KHz and 7 with losses in 0.5, 1, 2, and also in 4 KHz.

In the contralateral ear with normal otoscopy (paired control), the air pathway was normal in 100 (86.9%) cases, mild in 13 (11.3%) and moderate in 2 (1.3%) cases.

Considering bone conduction, we observed 5 (4.3%) cases of mild SNHL isolated in 4 KHz.

Analyzing bone conduction hearing loss alone in 4 KHz, there was no statistically significant difference between the ears with SCOM and the normal contralateral ear (P=0.19; OR=2.04; CI=0.60-6.75). When we compared the alteration in the hearing threshold by bone conduction for the mean frequencies of 0.5, 1, 2 KHz in both groups, we did not see losses in the normal ear and in the ear with SCOM, moderate SNHL was seen in one case and severe to profound hearing loss in 6 cases.

As to the presence of cholesteatoma, it was seen in 37 (32%) and it did not happen to 78 (68%) cases. As to the cholesteatoma interference analysis in SNHL development, Table 2 shows the 15 cases observed in the group with SCOM, and the cholesteatoma was associated with bone conduction hearing threshold (p=0.01), in such a way that the SNHL was more intense in the group with SCOM without cholesteatomas.

**Table 2:** Sensorineural hearing loss in 115 patients with unilateral suppurative chronic otitis media (37 with and 78 without cholesteatoma) and normal contralateral ear (paired control).

Bone conduction threshold	Suppurative chronic otitis media			Contralateral
	With cholesteatoma N=37(%)	Without cholesteatoma N=78(%)	Total	N=115(%)
> or = 30 dB in 0.5, 1, 2 KHz (average)	1(2,7)	6(7,7)	7(6,0)	0
> or = 30 dB only in 4 KHz	6(16,2)	2(2,5)	8(6,9)	5(4,3)
P value	0,58	0,01	0,08	1,00

## DISCUSSION

In the present investigation, average hearing loss of 40 Db was higher than the one observed in other series of cases, of which average varied from 19 to 33 Db in the frequencies of speech^6^, ^15^. The longest duration of ear disease (12.4 years) up to the surgical intervention, with probable higher frequency of breakage in the ossicular chain could justify such difference^11^.

In the ear with SCOM, the frequency of SNHL was of 13% (15 cases). In a similar study, carried out with patients with a similar socio-economical status to those from the patients selected in the present study, SNHL was seen in 10% of the cases reviewed^12^. It is likely that SNHL associated with SCOM is higher in populations of lower socio-economic statuses^2^. Difficulties to access treatment with antibiotics, inadequate follow up of patients and worse hygiene and education situation could corroborate this hypothesis.

Disease duration does not correlate with SNHL (Table 1). In a study with similar methodology to ours, MacAndie & O’Reilly observed the same lack of correlation, however they did not report on the mean duration of the ear disease in their patients^9^. This lack of correlation observed in this study should be assessed with some questioning. Mean time of disease duration was of 12.4 years. Therefore, there was a predominance of cases of long standing ear disease, in such a way that this correlation between SNHL with a longer or shorter time of ear disease duration was limited, having seen that there were few cases with shorter times of disease duration. The correlation between SNHL and SCOM was previously shown by many authors^5^, ^11^, ^12^. Considering all the studies evaluated, including the one hereby presented, it is plausible to speculate that the disease duration time variation, socio-economic status and cholesteatoma present could explain the different results found. The association observed between SNHL and adjusted age reinforces the idea that time-related variables could influence the tendency for SNHL in patients with SCOM.

SNHL was seen in SCOM with and without cholesteatomas (Table 2). In many a study, SNHL associated with SCOM without cholesteatoma was disregarded or little considered^5^, ^6^, ^7^, ^9^. It is worth mentioning that in these studies we considered cases of chronic otitis media that had their otorrhea interrupted with the antibiotic treatment. The cases evaluated in the present investigation included chronic otorrhea that did not resolve with the antibiotic treatment. The cases assessed in the present investigation included chronic otorrhea that did not stop after antibiotic treatment. The assessment of SNHL in ears with chronic infection with persistent and not-persistent otorrhea must be different. Toxins produced by bacteria present in a chronic purulent secretion in the middle ear would cause more damage to the inner ear when compared to non-persistent otorrhea and the toxins production is not continuous^16^, ^17^. Paparella et al. showed, in an elegant experimental study this association between SNHL and SCOM^18^. They emphasized the deleterious consequences of chronic otorrhea for the inner ear. The cochlear damage has been attributed to the passage of toxins through the round window, causing damage to the hair cells, especially in the cochlear base^16^, ^17^. Persistent inflammation associated with chronic otorrhea would increase the round window’s permeability to bacterial toxins^17^, ^18^. SNHL occurring in non-cholesteatomatous SCOM must be considered in the analysis of patients in whom surgical treatment is delayed for whatever reason. The explanation for a higher SNHL intensity in cases without cholesteatoma observed in the present investigation is merely speculative: the presence of an epithelial tumor would cause middle ear mucosal thickening, making it difficult for the bacteria to access the inner ear.

## CONCLUSION

According to the present study, SCOM with or without cholesteatoma may evolve towards SNHL, which was observed in 13% of the cases and is correlated with an age increase, but not with the presence of cholesteatoma or with a longer duration of otologic disease.
